# The power of evolutionary rescue is constrained by genetic load

**DOI:** 10.1111/eva.12489

**Published:** 2017-05-26

**Authors:** Gavin S. Stewart, Madeline R. Morris, Allison B. Genis, Marianna Szűcs, Brett A. Melbourne, Simon J. Tavener, Ruth A. Hufbauer

**Affiliations:** ^1^ Department of Mathematics Colorado State University Fort Collins CO USA; ^2^ Department of Mathematics Courant Institute of Mathematical Sciences New York NY USA; ^3^ Department of Biomedical Sciences Colorado State University Fort Collins CO USA; ^4^ Department of Biology Colorado State University Fort Collins CO USA; ^5^ Department of Bioagricultural Sciences and Pest Management Colorado State University Fort Collins CO USA; ^6^ Department of Ecology and Evolutionary Biology University of Colorado Boulder CO USA; ^7^ Graduate Degree Program in Ecology Colorado State University Fort Collins CO USA; ^8^ Centre de Biologie pour la Gestion des Populations (INRA, Montpellier SupAgro) Montferrier‐sur‐Lez Cedex France

**Keywords:** adaptation, evolutionary rescue, experimental evolution, genetic load, genetic rescue, inbreeding, population dynamics, population ecology

## Abstract

The risk of extinction faced by small isolated populations in changing environments can be reduced by rapid adaptation and subsequent growth to larger, less vulnerable sizes. Whether this process, called evolutionary rescue, is able to reduce extinction risk and sustain population growth over multiple generations is largely unknown. To understand the consequences of adaptive evolution as well as maladaptive processes in small isolated populations, we subjected experimental *Tribolium castaneum* populations founded with 10 or 40 individuals to novel environments, one more favorable, and one resource poor, and either allowed evolution, or constrained it by replacing individuals one‐for‐one each generation with those from a large population maintained in the natal environment. Replacement individuals spent one generation in the target novel environment before use to standardize effects due to the parental environment. After eight generations we mixed a subset of surviving populations to facilitate admixture, allowing us to estimate drift load by comparing performance of mixed to unmixed groups. Evolving populations had reduced extinction rates, and increased population sizes in the first four to five generations compared to populations where evolution was constrained. Performance of evolving populations subsequently declined. Admixture restored their performance, indicating high drift load that may have overwhelmed the beneficial effects of adaptation in evolving populations. Our results indicate that evolution may quickly reduce extinction risk and increase population sizes, but suggest that relying solely on adaptation from standing genetic variation may not provide long‐term benefits to small isolated populations of diploid sexual species, and that active management facilitating gene flow may be necessary for longer term persistence.

## 1 INTRODUCTION

It has become clear in the past decade that evolution can occur rapidly enough to influence ecological dynamics (Lowe, Kovach & Allendorf, 2017; Pelletier, Garant, & Hendry, 2009; Reznick & Ghalambor, 2001; Schoener, 2011; Turcotte, Reznick, & Hare, 2011). The power of the interplay between ecological and evolutionary processes, or eco‐evolutionary dynamics, has prompted a call for “evolutionarily enlightened management” in conservation biology (Ashley et al., 2003). Indeed, evolutionary principles are being increasingly applied to problems in conservation (Frankham, 2015; Hendry et al., 2011; Weeks et al., 2011).

One of the major challenges land and wildlife managers face is warding off extinction of small populations subjected to rapidly changing environments due to climate change, habitat loss and fragmentation, and pollution. Genetic rescue, an increase in population fitness owing to immigration of new alleles (Whiteley, Fitzpatrick, Funk, & Tallmon, 2015), is one of the primary evolutionarily based management approaches used to slow or stop the decline of dwindling populations. When migrants cross with residents, heterozygosity increases, masking deleterious mutations, and facilitating adaptive evolution (Frankham, 2016; Hedrick & Garcia‐Dorado, 2016; Hufbauer et al., 2015; Tallmon, Luikart, & Waples, 2004; Weeks et al., 2011; Whiteley et al., 2015). However, some populations may be able to adapt to changing environments from standing genetic variation without the aid of migrants (Carlson, Cunningham, & Westley, 2014; Gomulkiewicz & Holt, 1995; Hufbauer et al., 2015), which falls under the rubric of evolutionary rescue. Evolutionary rescue has been defined as genetic adaptation that allows population recovery from environmentally induced demographic effects that otherwise would have caused extinction (Carlson et al., 2014). Clearly, adaptation to a challenging environment can be facilitated by gene flow (Whiteley et al., 2015), but we use the term evolutionary rescue here in its strictest sense as evolution from standing genetic variation without migration (Hufbauer et al., 2015). Understanding the circumstances under which evolutionary rescue is likely to take place, as well as the limitations of evolutionary rescue, will help focus scarce resources on populations and species where more active management is necessary.

Population size and the degree of mismatch with the environment are two of the most important factors influencing evolutionary rescue. When populations are small, stochastic processes can increase the probability of extinction (Gomulkiewicz & Holt, 1995). If an environmental change leads to a mismatch between a population and its environment that reduces fitness, population sizes can decline rapidly, enhancing the role of stochasticity relative to adaptive evolution (Carlson et al., 2014; Gomulkiewicz & Holt, 1995). Thus, an important avenue of study would be to test how small population size and degree of mismatch with the environment together influence the probability of evolutionary rescue.

Much of the research on evolutionary rescue has involved yeast and bacteria (e.g., Bell & Gonzalez, 2009, 2011), which are powerful model organisms given their ability to reproduce asexually, their small size (which makes it possible to conduct experiments with large populations), and their ability to be resurrected from frozen samples to serve as controls for comparison with evolving populations. However, exactly these advantages may constrain our ability to apply findings to management of rare, threatened and endangered species, many of which are obligately sexually reproducing diploids.

In large populations of diploid species with obligate sexual reproduction, recessive deleterious mutations are often masked by a more favorable dominant allele, and thus have little effect on fitness. When such populations are small, both inbreeding and genetic drift can increase homozygosity at loci with deleterious alleles, reducing fitness and contributing significantly to extinction risk (Frankham, 2005a, 2005b; O'Grady et al., 2006). The difference in the mean fitness of a population and the fitness of an optimal genotype that does not carry deleterious mutations is called the genetic load (Glémin, Ronfort, & Bataillon, 2003; Kirkpatrick & Jarne, 2000). As a population loses variation via drift and inbreeding, genetic load increases. Genetic load tends to be lower in haploid organisms or those that have a life stage or sex that is haploid, because deleterious alleles are often exposed to selection rather than being hidden behind a favorable dominant allele as in diploids (Henter, 2003). Hence, an important step in research on evolutionary rescue is to better understand both its power and limits in obligately sexual diploid species that can experience high genetic load.

Two of the major components of genetic load are drift load and inbreeding load (Paland & Schmidt, 2003). Inbreeding load (essentially inbreeding depression) is measured by comparing the fitness of offspring resulting from mating between relatives to the fitness of offspring produced by random mating of individuals from the same population. Drift load can be estimated by comparing the fitness of individuals produced by outcrossing between populations to the fitness of offspring produced by random mating of individuals from one of those populations (Paland & Schmidt, 2003). When such outcrossed individuals have higher fitness, they are said to exhibit heterosis. Drift load is not strictly the flipside of heterosis (Glémin et al., 2003) because the outcrossed individuals are not free of deleterious alleles, but heterosis provides a conservative estimate of the magnitude of drift load. Inbreeding load and drift load are produced by the same deleterious mutations, and are conceptual bins related to how the effects of those mutations are measured. Inbreeding load shifts to drift load as a population loses heterozygosity (Keller & Waller, 2002).

To gain insight into the eco‐evolutionary dynamics of sexually reproducing diploid organisms we evaluated population dynamics over eight generations in populations of the red flour beetle (*Tribolium castaneum*). We founded beetle populations into two novel environments, both of which reduced population growth rates relative to their natal environment, and thus posed an evolutionary challenge. Evolving populations could adapt to the novel environments but also could experience nonadaptive processes such as inbreeding and genetic drift. To isolate the effects of evolutionary processes, we constrained evolution in control populations by replacing experimental individuals one‐for‐one each generation (after raising them for one generation in the target environment to standardize maternal effects). This approach allowed demographic fluctuations to occur without constraint, but prevented adaptation to the novel environments, and minimized inbreeding and genetic drift, as replacements originated from a large, well‐mixed stock population. We investigated how rapid evolution influenced extinction risk, population size, and population growth rate, and estimated the magnitude of drift load in evolving populations.

## 2 METHODS

Experimental populations came from a wild population of *T. castaneum* collected in Indiana, which had been reared in the laboratory for about 30 generations at the time of this experiment (the “SF” strain; Szűcs, Melbourne, Tuff, & Hufbauer, 2014; Hufbauer et al., 2015). Stock populations were maintained at 31°C at an average of 54% relative humidity on a standard medium of 95% wheat flour and 5% brewer's yeast, by weight. Beetles were reared in large populations of at least 1,000 individuals, in nonoverlapping generations lasting 35 days, following Melbourne and Hastings (2008), which maintained heterozygosity at microsatellite loci (Table S1). This controlled life history models organisms with discrete generations such as annual plants, many species of insect and fish.

Individuals founding our experimental populations were introduced to one of two novel, challenging environments in which population growth rates were expected to be substantially lower than the natal environment (Szűcs, Melbourne, Tuff, Weiss‐Lehman, & Hufbauer, 2017). One environment was relatively favorable (henceforth in this study called “favorable”) and the other environment was relatively poor (henceforth in this study called “poor”), with fewer resources. The novel aspect of both environments was the main source of carbohydrate: corn rather than the standard wheat flour. The favorable environment consisted of a mixture of 98.2% corn flour and 1.71% organic wheat flour and 0.09% brewer's yeast, while the poor environment consisted of 99.8% corn flour, 0.19% organic wheat flour, and 0.01% brewer's yeast (Table S2). The favorable environment was chosen to present a challenge (lower growth rates than in standard media) but not to be so harsh as to lead to a deterministic decline to extinction (Szűcs et al., 2017). In contrast, the poor environment, with nine times less of the most nutritious ingredient (brewer's yeast) than the favorable environment, was designed to be quite challenging, with extinction likely unless populations could adapt. Experimental populations were initiated across two temporal blocks to increase replication.

We initiated experimental populations at two founding sizes (*N*_0_ = 10 and 40). Each founding size is on a scale of immediate conservation concern (Lande 1993), with *N*_0_ = 10 being more likely to experience stochastic extinction, and likely to lose variation to drift and inbreeding more quickly. Experimental populations were initiated and maintained in 4 × 4 × 6 cm boxes containing 15 g of media. The founders were allowed 24 hr to mate and lay eggs. We did not manipulate sex ratio in the founders, and thus, particularly for *N*_0_ = 10, the sex ratio was likely to vary stochastically from 1:1. Females are polyandrous in this species, and exhibit last‐male sperm precedence in fertilization of eggs. Thus, the eggs that were laid by the founders could have represented genetic contributions from somewhat more (from multiple mating) or somewhat fewer (if not all individuals mated) than the 10 or 40 founders. This stochasticity in sex ratio and genetic contributions of founders add to both the realism and the variation in the dataset. After 24 hr, the founding adults were removed and discarded, leaving the eggs behind to develop into the next generation of adults over a period of 5 weeks. The new adults that emerged were placed on fresh medium and also allowed 24 hr to mate and lay eggs, and this procedure was repeated for eight generations. Using fresh medium each generation, wastes could not accumulate and resource availability remained consistent generation‐to‐generation. We censused all populations each generation.

In evolving populations, the beetles that reached adulthood within 5 weeks were used to found the next generation. Nonevolving populations, in contrast, were censused and then individuals were replaced each generation, one‐for‐one, using beetles from the source population. The source population was maintained at large size (1,000 individuals) on the standard wheat media, and thus could not adapt to the novel environments. Given the large size of the source population, genetic drift and inbreeding should be minimal relative to the experimental populations over the course of our experiment (Hartl & Clark, 2007). Because maternal effects are strong in *T. castaneum* (Hufbauer et al., 2015), replacement beetles spent one generation on the appropriate experimental environment (favorable or poor) prior to use. This allowed us to standardize maternal effects while providing little opportunity for long‐term adaptation to the novel environments. This does not allow us to rule out potential different epigenetic inheritances (Richards, Bossdorf, & Pigliucci, 2010; Skinner, 2015), given the difference in the environment of the grandparental generation, and the unique population densities experienced by the parental generation. Hereafter, we call the treatment in which evolution is constrained the control.

By generation 8, the size and growth rates of most evolving populations had declined to similar levels as those of the control populations (see Section 3). We hypothesized that inbreeding and genetic drift could have increased homozygosity over the course of the experiment, thereby increasing genetic load, and largely overwhelming the beneficial effects of adaptation.

To test our hypothesis that the drop in fitness in the second half of the multigeneration experiment was due to high genetic load, we facilitated outcrossing in generation 8 in a subset of the evolving populations by mixing individuals among populations and comparing their performance to unmixed evolving populations. Heterosis in individuals produced via admixture would suggest that deleterious recessive alleles were masked with outcrossing. The difference in fitness between mixed and unmixed populations provides an estimate of the effect of drift load (Paland & Schmidt, 2003), a main component of the total genetic load. We did not estimate inbreeding load, the other main component of the total genetic load (which would have required mating between relatives). In the context of our experiment, we can also provide an estimate of the magnitude of the effect of adaptation on fitness by comparing the performance of mixed evolving populations to control populations. Specifically, both mixed evolving populations and control populations should have low homozygosity and thus low drift load, the former due to recent outcrossing and the latter due to large population size and outcrossing every generation in the source population that control individuals were drawn from. A crucial difference between them is that mixed evolving populations had the opportunity to adapt while control populations did not. In common environment experiments, such as this, the classic interpretation is that differences in phenotype (in this case between mixed evolving and control populations) reveal underlying genetic differences (Clausen, Keck, & Hiesey, 1940). However, an alternative, nonmutually exclusive, explanation of differences in phenotype is that they are due to epigenetic effects, which can persist multiple generations (Richards et al., 2010). In the mixing experiment, the parental environment was the same, with the exception of some differences in beetle density, but the grand parental environment differed between evolving and control populations. Thus differences in fitness may also arise from trans‐generational environmental differences.

We had sufficient populations from the favorable environment to implement all three treatments in this experiment: unmixed evolving populations, mixed evolving populations, and control populations (in which mixing occurred every generation). In the poor environment, we had limited extant populations within each temporal block in the 8th generation (block 1, 7 evolving populations from *N*_0_ = 10 and 7 from *N*_0_ = 40; block 2, 11 evolving populations from *N*_0_ = 10 and 10 from *N*_0_ = 40). Given these low numbers, we implemented only two treatments in the poor environment: mixed evolving populations and control populations. By mixing all evolving populations together rather than just a subset, we improved the opportunity of increasing heterozygosity. In this way, the mixing treatment was relatively comparable between populations evolving within the favorable and the poor environments. Thus, in the poor environment, we were restricted to comparing mixed evolving populations to control populations, which estimates the magnitude of adaptation unencumbered by drift load rather than drift load per se (which would have required comparison between mixed and unmixed evolving populations).

Mixing occurred across founding sizes within media due to the small number of extant populations founded at small size in the poor environment within individual temporal blocks. Thus, with this experiment we can estimate overall effect size of heterosis and adaptation, by evolution treatment but not by founding population size. This likely increases the variability in the results, but should not alter the direction of the response if drift load is the cause of the lower performance of evolving populations in the final generations. Specifically, to perform the mixing, individuals from the appropriate groups of evolving populations were placed together in a single container after censusing in generation 8. These individuals were then used to continue the experimental populations through generation 9, providing a full generation for outcrossing to occur among individuals from formerly separated populations. Population growth rate was then measured in generation 10.

### 2.1 Statistical analyses

To understand the consequences of rapid evolution for extinction in challenging environments, we evaluated whether the probability of extinction over the course of the experiment varied by evolution treatment. There was only one extinction in the favorable environment—in a control population initiated at a founding size of *N*_0_ = 10. Thus, there was no variance in extinction among three treatments in the favorable environment (both founding sizes of evolving populations, and large nonevolving populations) and a full, generalized linear mixed model (GLMM) including environment could not be fitted (the separation problem, Albert and Anderson 1984). To be able to fit the full model, we used a simple data augmentation approach in which we added one trial with extinction to the dataset for the three affected treatment combinations. The full model of extinction probability used a binomial distribution and a logit link (SAS Institute, Inc 2008), and included initial population size (a categorical variable with two levels, small and large), and evolution treatment (evolving or control), and environment (favorable or poor), and all their interactions. Block was included as a fixed effect, as there were only two temporal blocks. The augmentation approach is known to have problems as an all‐purpose method, particularly when replication is low (Agresti & Yang 1987; Heinze & Schemper 2002). To evaluate its performance, we compared results from the full model with the augmented dataset to a reduced model that excluded the affected treatment combinations. The relative estimated probabilities of extinction among treatment combinations were the same to ±0.03, and estimated confidence interval widths differed by <2%. The augmented dataset approach thus performed quite satisfactorily in this case with large sample size.

For the treatment combinations with no extinction, we took the estimated probability and one side of the confidence limit to be zero (negligibly different from the estimated confidence limit in practice) and drew the upper confidence limit from the augmented data analysis. As a further check of these confidence limits, we also calculated exact 95% confidence widths for the affected treatment combinations as 1 − exp(ln(0.05)/*n*), where *n* is the number of trials, assuming that trials are independent (in contrast the GLMM does not assume independence). The exact intervals were slightly smaller than the intervals estimated by the GLMM using the augmented dataset, suggesting that the estimated GLMM intervals are conservative, with the full model accounting for extra variation. The unaugmented dataset was used to estimate *p*‐values for the main effects of genetic background and founding size (as the separation problem did not occur for the main effects), while the augmented dataset was used to estimate the *p*‐values for their interactions. We estimated odds ratios and their confidence intervals to examine differences between treatments.

We also evaluated the time it took for those populations that went extinct to go extinct, focusing on populations from the poor environment. Time to extinction (in generations) was log transformed prior to analysis to improve the normality of residuals. Factors in the model were founding size and evolution treatment, with block as a fixed effect.

One of the most important characteristics of populations that managers track is population size. In our analyses of population size, we focused on populations that persisted to the end of the experiment. We evaluated the influence of evolution treatment, founding size, environment, generation, and their interactions on population size in a repeated measures linear mixed model that included temporal block and population size in the previous generation as fixed effects, with individual populations being the units on which measures were repeated. Effects reported for this model, population growth rate, and the mixing experiment, are from type III sums of squares (SAS 9.2, SAS Institute, Inc 2008). Population size was log transformed prior to analysis to improve the normality of the residuals, and back‐transformed results are shown.

We used the same model that we used for population size to analyze population growth rates (*N*_*t*_/*N*_*t*−1_). As for the analyses of population size, we included block as a fixed effect, and log transformed the growth rates prior to analysis to improve the normality of the residuals. To test our hypothesis that high genetic load reduced performance of the closed evolving populations, we compared the growth rate of evolving populations that had been mixed together to the growth rate of control populations using a mixed model of population growth (*N*_*t*_/*N*_*t*−1_) that included environment, treatment, media, and population size, with block as a fixed effect. For a visual comparison of performance of evolving populations in the poor environment to the mixed populations, we also graph population growth from the generation prior to mixing (evo* in Figure 4).

## 3 RESULTS

### 3.1 Extinction

**Figure 1:**
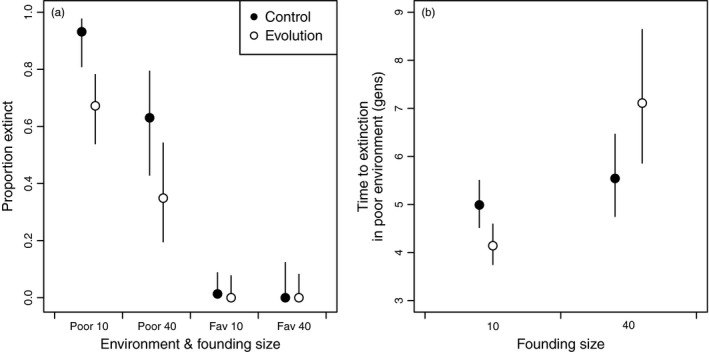
(a) The proportion of populations that went extinct over the course of the experiment. Means and 95% confidence intervals are from the model (see Statistics for details). In the favorable environment, extinction did not occur, except in one small, control population. (b) Mean time to extinction (with 95% confidence intervals), focusing on populations that went extinct, for populations maintained in the poor environment only, illustrating the significant interaction in the model between evolution treatment and founding size

More than half of the populations went extinct in the poor environment and only a single control population founded with 10 individuals went extinct in the favorable environment (Figure 1a, Figures S1 and S2). Environment, founding size, and evolution treatment all influenced extinction, and no interactions between these main effects were significant (Table S3). The odds of extinction for populations with 10 founders was 4.9 times greater (95% CI: 2.2, 10.9) than the odds of extinction for populations with 40 founders (Tables S2 and S3). The odds of extinction of populations that were not allowed to adapt was 4.6 times greater (95% CI: 2.0, 10.5) than the odds of extinction for the evolving populations (Tables S2 and S3). The evolution treatment reduced odds of extinction to similar extents at both founding sizes compared to the controls (nonsignificant evolution treatment × founding size interaction term, Table S2).

### 3.2 Time to extinction

Focusing only on the poor environment, in which extinctions were common, populations with a smaller initial size went extinct more quickly than those starting at a larger size (*F*_1, 98_ = 19.83, *p *<* *.0001, Figure 1b). While there was not a significant main effect of the evolution treatment (*F*_1, 98_ = 0.2, *p *=* *.65), there was an interaction between treatment and founding size (*F*_1, 98_ = 9.08, *p *=* *.0033, Figure 1b), such that in populations founded at small size, extinction was 1.2 times faster in evolving populations than control populations (95% CI: 1.05, 1.38). At the same time in initially larger (*n* = 40) evolving populations extinction was 1.2 times slower than in control populations (95% CI: 1.00, 1.64) or in populations founded at small size (Figure 1b). In small populations, the apparent detrimental effect of evolution on time to extinction could be driven by the intensity of the bottleneck at founding. Evolving populations founded with only 10 individuals would become homozygous quickly, and genetic load could then offset the beneficial effects of adaptation to the environment more quickly for small than for large populations. Additionally, beneficial grand‐maternal effects (van Allen & Rudolf, 2013) might have contributed to maintaining relatively higher fitness of control populations in the poor environment compared to evolving populations, stabilizing their populations.

### 3.3 Population size

**Figure 2:**
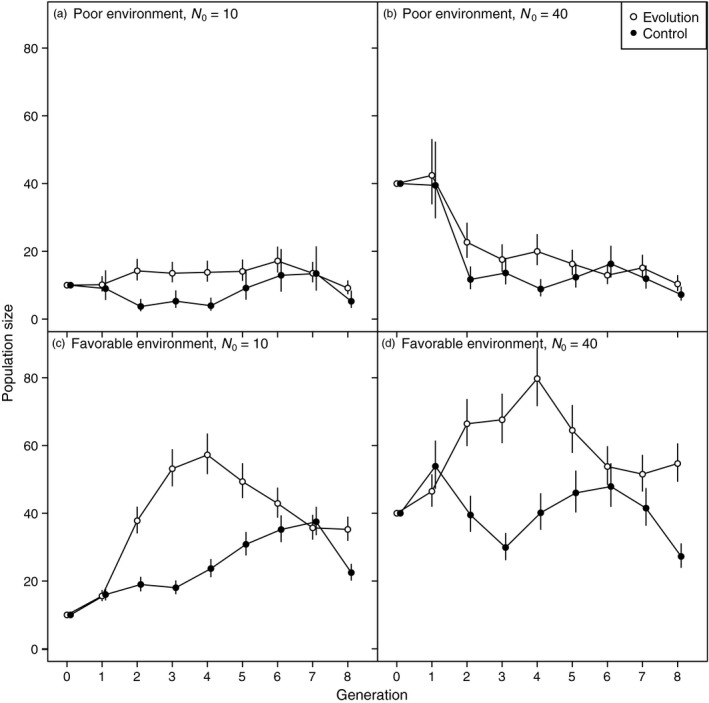
Population sizes through time for all treatment combinations, focused on populations that were extant at the end of the experiment. Means and 95% confidence intervals are back‐transformed from model estimates

In the poor environment, evolving populations maintained slightly higher sizes than the controls throughout the experiment (Figure 2a,b, Figure S1 and Table S4). Populations founded at the larger size quickly shrank to about the size attained by the smaller populations, reflecting the low carrying capacity of that environment.

In the favorable environment, the mean sizes of the evolving and control populations diverged rapidly between generations 1 and 4 for both the initially smaller and larger populations (Figure 2a,b). In these early generations, evolving populations increased in size more than controls in the favorable environment, and maintained higher size than controls in the poor environment, likely as a result of adaptation. Between generations 4 and 8, the sizes of the evolving populations decreased. This decrease is more evident in the favorable environment, where populations had initially grown rapidly, than in the poor environment.

### 3.4 Population growth rates

**Figure 3:**
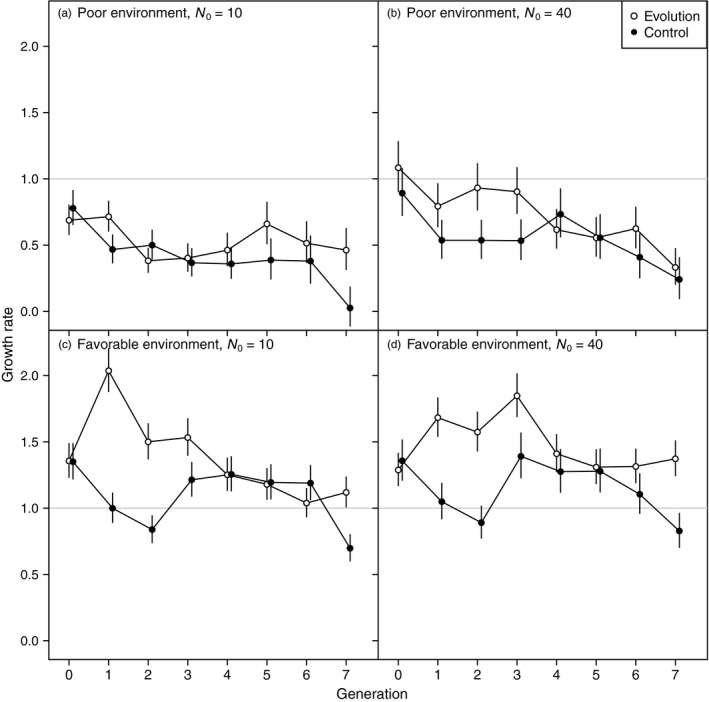
Density independent growth rates for each generation for all treatment combinations. Because generation 8 was the final census, growth data for generation 8 to generation 9 are not available. Means and 95% confidence intervals are back‐transformed from model estimates

The effects of evolution shifted through time (significant treatment × generation interaction, Table S5). Evolution initially caused growth rates to rapidly increase compared to control populations. In generations 2‐4, evolving populations grew faster than control populations, except for small populations in the poor environment (Figure 3). The clearest positive effect on growth rates was in populations founded at the larger size in the favorable environment. In later generations, this beneficial effect of evolution was lost. We hypothesize that decreased performance was due to increased homozygosity over time, which would increase genetic load (Figure 3). Small populations might be expected to have reduced performance earlier than large populations, as deleterious mutations should be fixed more quickly. There is no evidence of this in the favorable environment, but there is in the poor environment, where growth rates tended to be lower in the first four generations in populations founded by 10 individuals than in populations founded by 40 individuals (Figure 3a,b). The low growth of control populations in the final generation, particularly in the favorable environment, is unexpected, and likely attributable to a laboratory error in making the growth media.

### 3.5 Effects of admixture

**Figure 4:**
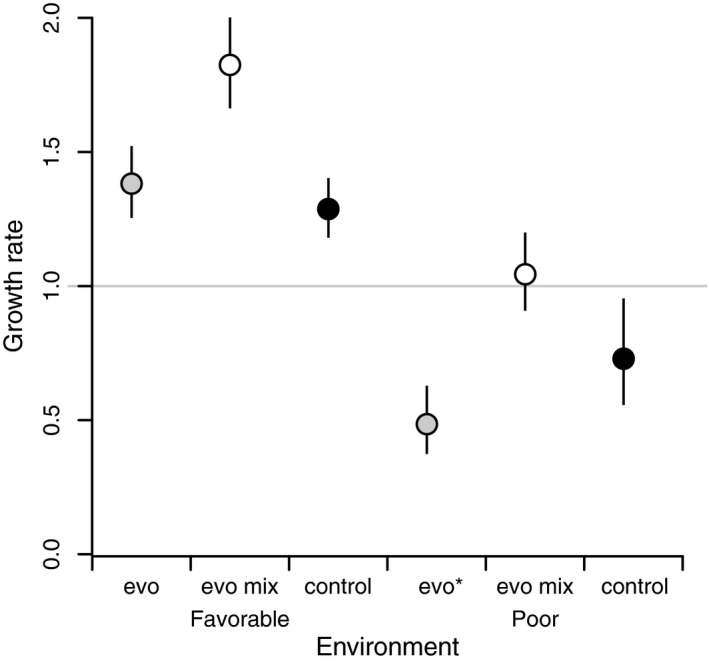
Growth rate of evolving populations that were mixed together for a single generation to alleviate genetic load (evo mix), control populations, and evolving populations that were not mixed together (evo). In the favorable environment, all three treatments were available (evo mix, evo, and control). In the poor environment, low numbers of extant populations required *all* evolving populations to be mixed. For visual comparison we provide the mean growth rate of unmixed evolving populations in the poor environment from the final generation of the experiment (*N*_8_/*N*_7_) (the “evo*” value)

Prior to mixing, the size and growth rate of evolving populations began to decline in both environments, suggesting that inbreeding and genetic drift had increased homozygosity and fitness was subsequently reduced (Figures 2 and 3). Mixing individuals from different populations together increased the growth rate in both environments (Figure 4). In the favorable environment, the difference between closed evolving populations and mixed evolving populations represents heterosis associated with outcrossing via the masking of drift load (effect size and CL in the favorable environment 0.35 [0.22, 0.47]). Mixed evolving populations also performed better than control populations (*F*_1,222_ = 20.82, *p *<* *.0001) in both environments. This comparison between mixed and control populations, neither of which should experience substantial drift load, estimates the strength of adaptation to the novel environment (effect size and CL in the poor and favorable environments respectively, 0.28 [0.17, 0.39]; 0.36 [0.10, 0.62], Figure 4). Interestingly, the growth rate of the mixed evolving populations was positive even in the poor environment, while growth of the control populations remained negative.

## 4 DISCUSSION

We show that evolution in small populations can reduce extinction rates in novel, challenging environments, even in those with a relatively high degree of mismatch and maladaptation such as in our poor environment. Moreover, we found that evolution can increase population sizes and growth rates over populations that are not permitted to adapt to the environment. However, evidence suggests that with time, genetic load, including drift load, and inbreeding load, may accumulate in small populations reducing initial higher growth rates achieved by adaptive evolution.

Small populations in a poor or stressful environment have a high probability of extinction (Frankham, 2005a, 2005b; Lopez, Rousset, Shaw, Shaw, & Ronce, 2009; Matthies, Brauer, Maibom, & Tscharntke, 2004; de Vere, Jongejans, Plowman, & Williams, 2009). Our results reconfirm this pattern, and reveal that those high extinction rates would likely be higher without the beneficial effects of adaptive evolution. We found that even under our extreme conditions of small founding sizes (10 individuals) in a poor environment, evolution reduced the extinction rate dramatically—from 93% in control populations to 67% in evolving populations over the course of eight generations. Larger populations (founded with 40 individuals) had a lower extinction rate than smaller ones, even though they could not grow due to the low carrying capacity of the poor environment. Nonetheless, evolution in the larger populations also reduced the rate of extinction—from 59% in control populations to 37% in evolving populations.

In the favorable environment, in contrast, there was almost no extinction: Only a single control population (*N*_0_ = 10) went extinct. Both the evolving and control populations grew rapidly in this environment (Figure 3), reducing their risk of extinction due to demographic stochasticity (Lande 1993). The finding that control populations could persist as well as evolving populations if the environment was relatively favorable (though still reducing fitness) is important, as it highlights the tremendous importance of habitat quality in conservation.

Evolution led to increased growth rates in the first few generations. High growth rates and larger population size were particularly marked in the novel environment that posed a mild challenge (our favorable environment). Evidence suggests that the reduced extinction and improved performance of evolving populations was due to adaptation to the novel corn environments. *Tribolium castaneum* can adapt to corn in several ways, including changes in larval food preference and reduced adult body size (Agashe, Falk, & Bolnick, 2011).

The advantages associated with evolution disappeared by about seven generations after founding. Following early rapid growth, evolving populations began to decline in generation 5, and eventually reached sizes similar to those of control populations. We hypothesize that the drop in performance is due to nonadaptive genetic processes that are known to impact small populations, such as inbreeding and genetic drift, which would have increased genetic load over time in our evolving populations. Using these same populations, Szűcs et al. (2017) also found evidence for genetic load in the form of inbreeding depression (offspring from consanguineous mating had lower fitness than offspring from random mating). Strong selection on traits conferring adaptation could have reduced overall genetic diversity and increased homozygosity in our experimental populations, and at the same time, genetic drift due to small population size could also have contributed to the rapid fixation of deleterious alleles. We propose that this created a situation in which populations were burdened with high genetic load, which reduced population fitness and effectively reversed evolutionary rescue. Our hypothesis is supported by the results from our mixing experiment, in which mixed populations exhibited heterosis. The only change experienced by the mixed populations was the opportunity to mate with individuals from different populations. As the evolving populations had been isolated from each other, genetic drift and inbreeding could have fixed different deleterious alleles, and crossing between them would serve to mask those alleles. Indeed, it is well documented that crossing populations can alleviate inbreeding even if those populations are inbred themselves (Coutellec & Caquet, 2011; Frankham, 2005a, 2005b; Hedrick & Garcia‐Dorado 2016). However, we do not have molecular genetic data to document that heterozygosity decreased over time or increased with mixing. Interestingly, in an experiment focused on different questions but also using *T. castaneum* and a corn environment, Falk, Parent, Agashe, and Bolnick (2012) observed a similar pattern: initial evidence of adaptation (reported in Agashe et al., 2011), which several generations later was followed by decreased fitness. Falk et al. (2012) also propose that the decrease in fitness was likely due to fixation of deleterious alleles.

The environments provided did not otherwise change during the mixing experiment, and thus the difference between mixed and unmixed populations indicates heterosis. Mixture resulted in population growth (Figure 4) even in the poor environment where otherwise evolving and control populations were declining. This suggests that populations in the poor habitat likely would have been able to achieve a positive growth rate via adaptation had they not been constrained by genetic load. This interpretation of our findings fits both theoretical (Lopez et al., 2009) and other empirical (Schleuning, Niggermann, Becker & Matthies, 2009) results, showing that isolation can rapidly increase genetic load and constrain population performance.

Comparisons of mixed evolving populations with control populations provide evidence for the power of adaptation. Interestingly, drift load and adaptation had effects on population growth rates of comparable size but of opposing direction, such that the beneficial effects of adaptation were essentially entirely obscured by the detrimental effects of genetic load.

An alternative interpretation of the comparison between mixed evolving populations and control populations is that differences in performance could be due to differences in the environment of the grandparents of the individuals in the experiment. We see two main ways this could occur. First, epigenetic changes, such as altered DNA methylation, that increase performance in the novel environment but take more than one generation to emerge could lead to a pattern of increased fitness in evolving populations but not in control populations. This is unlikely to explain our findings, as the increased performance relative to control is only seen in the mixed evolving populations, not the evolving populations that were not mixed. Second, a high‐quality grand‐maternal environment is known to improve performance of *T. castaneum* (van Allen & Rudolf, 2013), and thus the nutrient rich grand‐maternal environment of individuals in the control population could lead to high performance without adaptation—this would create a conservative bias reducing the magnitude of the difference we measured between mixed evolving populations and control populations. The difference was substantial nonetheless. Thus, adaptation appears to be the most parsimonious explanation of the difference in fitness between the mixed evolving populations and the control populations.

Without both the nonevolving controls and the mixture experiment at the end, the potency of adaptive evolution and genetic load in driving population growth and decline would not be clear. Indeed, the increase in population size followed by the decrease in the favorable environment might be interpreted solely as negative density dependence leading to fluctuation about a carrying capacity, were it not for the control populations providing the relevant comparison. Similarly, the minor increases in population size in the evolving populations in the poor environment might be interpreted as lack of adaptation, were it not for evidence of adaptation from the mixture experiment. These results from the poor environment support the idea that eco‐evolutionary dynamics can be quite cryptic (Kinnison, Hairston, & Hendry, 2015), even when evolving populations are compared directly to nonevolving controls, and challenge us to reexamine the limits to adaptation in small populations (Willi, Van Buskirk, & Hoffmann, 2006).

Our findings fit well with theoretical work suggesting that recombination may be important in evolutionary rescue (Uecker & Hermisson, 2016), as well as the growing body of literature on the role of on‐going migration in maintaining genetic variation and reducing inbreeding depression (Carlson et al., 2014; Whiteley et al., 2015). Our data support the idea that admixture masked deleterious mutations in our experimental populations. Other work also confirms a role for admixture or hybridization, or simply sexual reproduction and recombination, in providing genetic variation on which selection can act to fuel evolutionary rescue (Bell, 2013; Stelkens, Brockhurst, Hurst, & Greig, 2014; Uecker & Hermisson, 2016).

In sum, our results show that adaptation can reduce extinction even in very poor environments, and can drive population growth in better environments. However, the long‐term potential for adaptation to rescue isolated populations can be limited by increased homozygosity and genetic load. The speed at which fixation of deleterious alleles limits adaptation will clearly be determined by the starting level of heterozygosity and genetic load in natural populations. While our experimental populations were variable enough to adapt in this experiment (see also Hufbauer et al., 2015 and Szűcs et al., 2017) founders likely harbored less genetic variation than found in many natural populations, which could thus potentially adapt more quickly, and exhibit the negative effects of genetic load more slowly.

This study demonstrates clearly that evolution can rapidly alter ecological dynamics, but also highlights that evolution is a double‐edged sword—with both beneficial and deleterious processes powerfully shaping population size and performance. To manage the deleterious side, ongoing migration between otherwise isolated populations may be crucial for long term population health (Frankham, 2016), and the pros and cons of facilitating such migration should be considered carefully, especially if migrants are not adapted to the habitat (Fitzpatrick et al., 2016).

Hamilton and Miller (2016), explicitly provoke a conversation among conservation biologists struggling with weighing the importance of maintaining distinct evolutionary units relative to the importance of maintaining the evolutionary potential of populations to face the challenges presented in our changing world. We concur with them: Adaptive potential may help ensure population survival, and with reasonably low levels of gene flow, local adaptation may subsequently evolve anew, even if temporarily disrupted via gene flow. The risks of outbreeding depression may be exaggerated (Frankham et al. 2011) and the benefits of genetic rescue are potent (Frankham, 2015, 2016). Nonetheless, migration of nonadapted individuals should be facilitated only with caution and with previous study of the potential outcomes. Our results suggest that the apparent vigor of small populations coping with environmental change should not lead to complacency and no action, as adaptation may be transient and could be offset by genetic drift unless gene flow is actively promoted.

## DATA ARCHIVING STATEMENT

Data available from the Dryad Digital Repository: https://doi.org/10.5061/dryad.t3q41.
