# Supplementary material for: The power of evolutionary rescue is constrained by genetic load
Source: Evol Appl. 2017 May 26;10(7):731–41. doi: 10.1111/eva.12489 (PMC5511356; doi:10.1111/eva.12489)
Supplement: Supplementary file 1 [file EVA-10-731-s001.docx]

*Genotyping*

Genomic DNA was extracted using Agencourt DNAdvance kit following manufacturer`s protocol. The 9 loci used for genotyping were chosen from Demuth et al. 2007 based on levels of polymorphism in their test populations and whether they reliably amplified in our populations. We concentrated on tri-nucleotide repeats but one locus with dinucleotide repeats was also included. The 12 loci were run in four separate multiplex reactions with 2-4 loci in each (Table S1) using the Qiagen Multiplex PCR Kit and by adding the M13(-21) oligonucleotide adaptor sequence to the 5` end of the forward primers (Schuelke 2000). Microsatellites were amplified in 10.5-μL reactions consisting of 0.58x of Qiagen Multiplex PCR Master Mix containing: HotStarTaq DNA Polymerase, Multiple PCR buffer with 3mM Mg^2+^, and dNTP mix; 0.03 μM of each forward primer, 0.1 μM of each reverse primer, 0.2 μM (for multiplex with 2 loci), 0.3 μM (for multiplex with 3 loci), or 0.4 μM (for multiplex with 4 loci) of fluorescence-labelled (NED, PET, FAM or VIC) M13(-21) primer and with 50-100 ng of genomic DNA. For the PCR the following cycling conditions were used: 95$^{\circ}$C for 15 min to active the HotStarTaq, then 94$^{\circ}$C for 30 sec, 57$^{\circ}$C for 90 sec, 72$^{\circ}$C for 60 sec (25 cycles), 94$^{\circ}$C for 30 sec, 53$^{\circ}$C for 60 sec, 72$^{\circ}$C for 60 sec (8 cycles), 60$^{\circ}$C for 30 min. PCR products were diluted 10-fold, and then 0.6 μL of each sample with different fluorescent labels were combined with 0.3 μL of GENESCAN-500 LIZ Size Standard (Applied Biosystems) and 9.7 μL Hi-Di Formamide. Samples were covered with a foil lid, denatured at 90$^{\circ}$C for 15 min and genotyped on an ABI 3730. Alleles were scored using GENEMAPPER 3.7 (Applied Biosystems).

Table S1. Genetic diversity measures (mean and standard error) for the ‘SF’ lineage of *T. castaneum* over 9 microsatellite loci. Number of individuals (N), number of alleles (Na), observed heterozygosity (Ho), and unbiased expected heterozygosity (uHe) as calculated in GenAlEx 6.502.

| **Lineage** | **N** | **Na** | **Ho** | **uHe** |
| --- | --- | --- | --- | --- |
| SF | 29 | 4.44 (0.80) | 0.53 (0.08) | 0.59 (0.09) |

Table S2. Experimental design, replication, and raw numbers and proportion of populations that went extinct.

| Media | No. of founders | Treatment: No. replicates | No. of extinctions | Proportion extinct |
| --- | --- | --- | --- | --- |
| Rich: 98.2 |  |  |  |  |
|  | 10 | evolution: 84 | 0 | 0 |
|  |  | control: 71 | 1 | 0.014 |
|  | 40 | evolution: 81 | 0 | 0 |
|  |  | control: 49 | 0 | 0 |
| Poor: 99.8 |  |  |  |  |
|  | 10 | evolution: 57 | 37 | 0.649 |
|  |  | control: 47 | 40 | 0.851 |
|  | 40 | evolution: 28 | 10 | 0.357 |
|  |  | control: 28 | 16 | 0.571 |
|  | 10 | 104 | 82 | 0.788 |
|  | 40 | 56 | 26 | 0.464 |

Table S3. Analyses of extinction, with the unaugmented dataset providing results for the main effects, and the augmented dataset (with a trials with extinction added for each of the three treatment combinations that experienced no extinction) providing results for the interactions.

| Effect | df | F | Pr >f |
| --- | --- | --- | --- |
| Treatment (evolution, no-evolution) | 1, 428 | 13.14 | 0.0003 |
| N_0_ (10, 40) | 1, 428 | 15.16 | 0.0001 |
| Environment (favorable, poor) | 1, 428 | 43.74 | <.0001 |
| Treatment x N_0_ | 1, 427 | 0.04 | 0.8444 |
| Treatment x Environment | 1, 427 | 1.27 | 0.2606 |
| Environment x N_0_ | 1, 427 | 3.05 | 0.0813 |
| Treatment x Environment x N_0_ | 1, 427 | 0.22 | 0.6358 |

Table S4. Statistical results for population size, conditional on populations being extant at the final census. See text for details of analysis.

| Effect | df | F Value | Pr > F |
| --- | --- | --- | --- |
| N_(t-1)_ | 1,2303 | 83.68 | <.0001 |
| Temporal Block | 1,29 | 6.37 | 0.0173 |
| Treatment (evolution, no-evolution) | 1,295 | 80.31 | <.0001 |
| N_0_ (10, 40) | 1,295 | 89.57 | <.0001 |
| Environment (favorable, poor) | 1,295 | 511.29 | <.0001 |
| Generation | 7,2065 | 18.12 | <.0001 |
| Treatment x N_0_ | 1,295 | 3.41 | 0.0658 |
| Treatment x Environment | 1,295 | 0.07 | 0.785 |
| Treatment x Generation | 7,2065 | 23.73 | <.0001 |
| Environment x N_0_ | 1,295 | 0.02 | 0.8864 |
| Environment x Generation | 7,2065 | 25.8 | <.0001 |
| Generation x N_0_ | 7,2065 | 18.21 | <.0001 |
| Treatment x Environment x N_0_ | 1,295 | 1.62 | 0.2046 |
| Treatment x Environment x Generation | 7,2065 | 4.68 | <.0001 |
| Treatment x Generation x N_0_ | 7,2065 | 2.65 | 0.0099 |
| Environment x Generation x N_0_ | 7,2065 | 1.26 | 0.2649 |
| Treatment x Environment x Generation x N_0_ | 7,2065 | 1.03 | 0.4055 |

Table S5. Statistical results for population growth. See text for details of analysis.

| Effect | DF | F Value | Pr > F |
| --- | --- | --- | --- |
| N_(t-1)_ | 1,2819 | 471.35 | <.0001 |
| Temporal Block | 1,50 | 0 | 0.9506 |
| Treatment (evolution, no-evolution) | 1,418 | 94.32 | <.0001 |
| N_0_ (10, 40) | 1,418 | 6.49 | 0.0112 |
| Environment (rich, poor) | 1,418 | 700.7 | <.0001 |
| Generation | 7,2492 | 20.23 | <.0001 |
| Treatment x N_0_ | 1,418 | 2.21 | 0.1375 |
| Treatment x Environment | 1,418 | 3.73 | 0.054 |
| Treatment x Generation | 7,2492 | 19.45 | <.0001 |
| Environment x N_0_ | 1,418 | 0.02 | 0.8835 |
| Environment x Generation | 7,2492 | 7.13 | <.0001 |
| Generation x N_0_ | 7,2492 | 4.89 | <.0001 |
| Treatment x Environment x N_0_ | 1,418 | 0 | 0.9574 |
| Treatment x Environment x Generation | 7,2492 | 1.99 | 0.0524 |
| Treatment x Generation x N_0_ | 7,2492 | 1.06 | 0.3855 |
| Environment x Generation x N_0_ | 7,2492 | 2.87 | 0.0055 |
| Treatment x Environment x Generation x N_0_ | 7,2492 | 1.74 | 0.0944 |

Figure S1: Population sizes of initially small (n = 10) and larger (n = 40) populations through time in the resource poor environment before mixing. Each line represents a single replicate population. Black lines represent populations that survived to the end of the experiment, and red lines indicate populations that went extinct.

Figure S2: Population sizes of initially small (n = 10) and larger (n = 40) populations through time in the favorable environment before mixing. Each line represents a single replicate population. Black lines represent populations that survived to the end of the experiment, and red lines indicate populations that went extinct.
